# Laparoscopic radical right hemicolectomy for cecal cancer and middle colic artery aneurysm

**DOI:** 10.1186/s12957-015-0595-5

**Published:** 2015-05-06

**Authors:** Konosuke Moritani, Osamu Wada, Heita Ozawa, Shin Fujita, Kenjiro Kotake

**Affiliations:** Department of Surgery, Tochigi Cancer Center, 4-9-13, Yohnan, Tochigi-ken, Utsunomiya, 320-0834 Japan

**Keywords:** Laparoscopic surgery, Cecal cancer, Middle colic artery aneurysm

## Abstract

**Background:**

Middle colic artery (MCA) aneurysms are very rare and exclusively reported with symptoms or rupture. We report successful laparoscopic elective surgery for both cecal cancer and MCA aneurysm in an 87-year-old man who presented with bloody stools.

**Methods:**

Diagnostic colonoscopy revealed a cecal tumor 40 mm in diameter that was histologically confirmed as a well-differentiated adenocarcinoma. The three-phase dynamic computed tomography showed a cecal tumor without any metastasis and an MCA aneurysm 10 mm in diameter. Radical right hemicolectomy with D3 lymph node dissection that included the MCA aneurysm was performed. The postoperative course was uneventful, and the patient survived without recurrence.

**Conclusions:**

Even though the present patient was very elderly, the postoperative course of laparoscopic radical surgery for both an MCA aneurysm and cecal cancer was uneventful with good short-term outcomes.

## Background

The middle colic artery (MCA) branches from the superior mesenteric artery near the pancreatic head and supplies the transverse colon and, occasionally, the hepatic flexure [1]. Although the incidence of visceral artery aneurysm is extremely low, the most frequent site is the splenic artery [2]. To the best of our knowledge, there have been no reports of treatment for MCA aneurysm occurring simultaneously with cecal cancer, likely owing to the rare occurrence. Laparoscopic surgery has become a practical treatment option for colorectal cancer, and the safety and efficacy of this approach has been demonstrated in clinical trials [3]. Here, we report the successful use of laparoscopic surgery for very elderly patients with both cecal cancer and an MCA aneurysm.

## Case presentation

**Figure 1:**
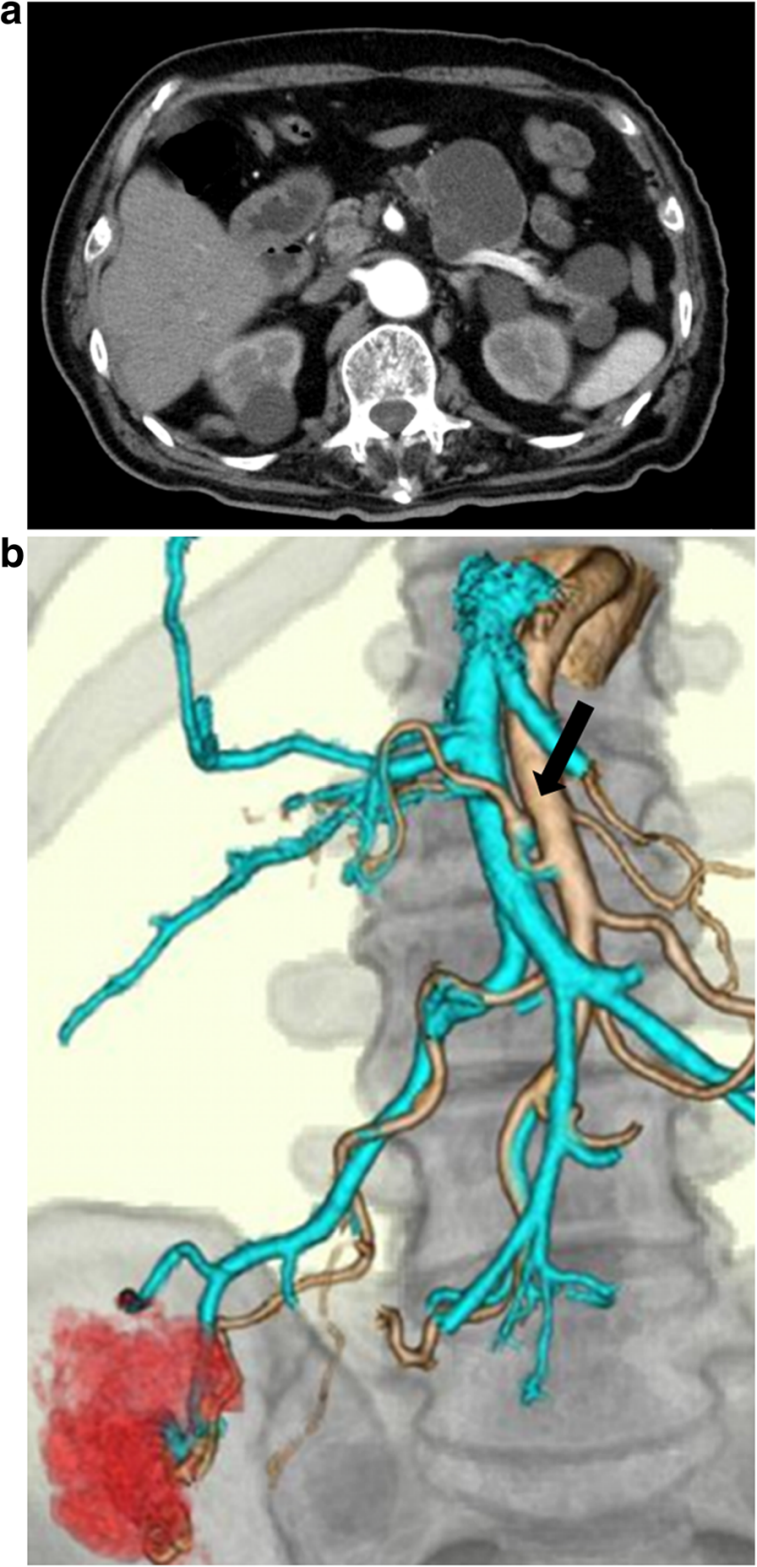
Preoperative computed tomography (CT) scan imaging **(a)** and CT angiography **(b)** showing a middle colic artery aneurysm (black arrow) measuring 10 × 7 mm without extravasation.

An 87-year-old man presented with a positive fecal occult blood test. He had no history of abdominal colic or distension. His medical history included hypertension and acute appendicitis that was resected 60 years previous. He had no family history of aneurysm, infectious disease, or autoimmune disorder. He quit smoking 30 years previously. His diagnostic colonoscopy revealed a cecal tumor of approximately 40 mm, and the biopsy specimen showed a well-differentiated adenocarcinoma. Preoperative three-phase dynamic computed tomography for disease staging showed a cecal tumor of 4 × 4 cm without metastasis and an MCA aneurysm of 10 × 7 mm (Figure 1).

**Figure 2:**
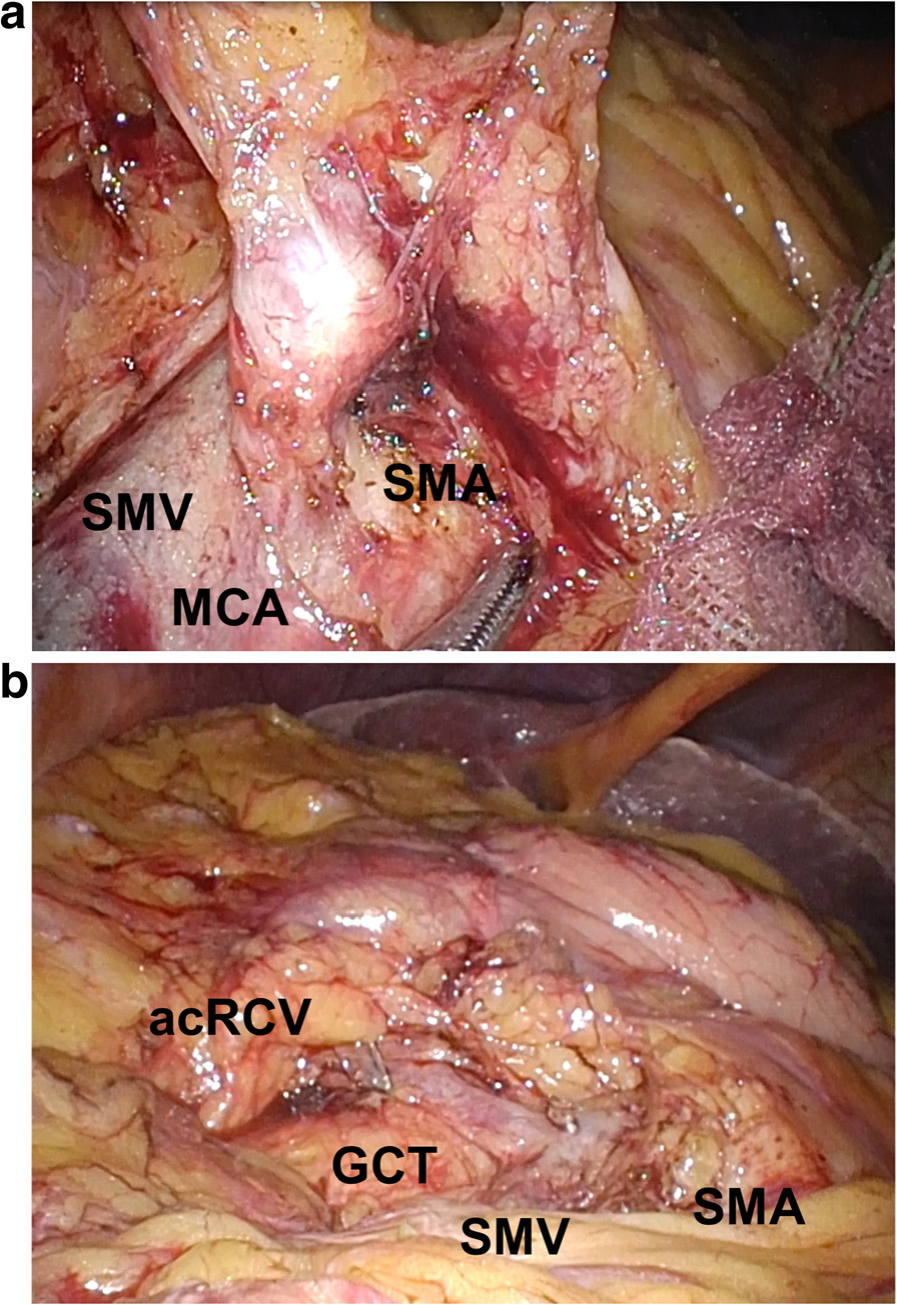
Laparoscopic view of a middle colic artery (MCA) aneurysm **(a)**. The MCA was ligated and divided at the root during radical right hemicolectomy with D3 lymph node dissection **(b)**. acRCV, accessory right colic vein; GCT, gastrocolic trunk; MCA, middle colic artery; SMA, superior mesenteric artery; SMV, superior mesenteric vein.

**Figure 3:**
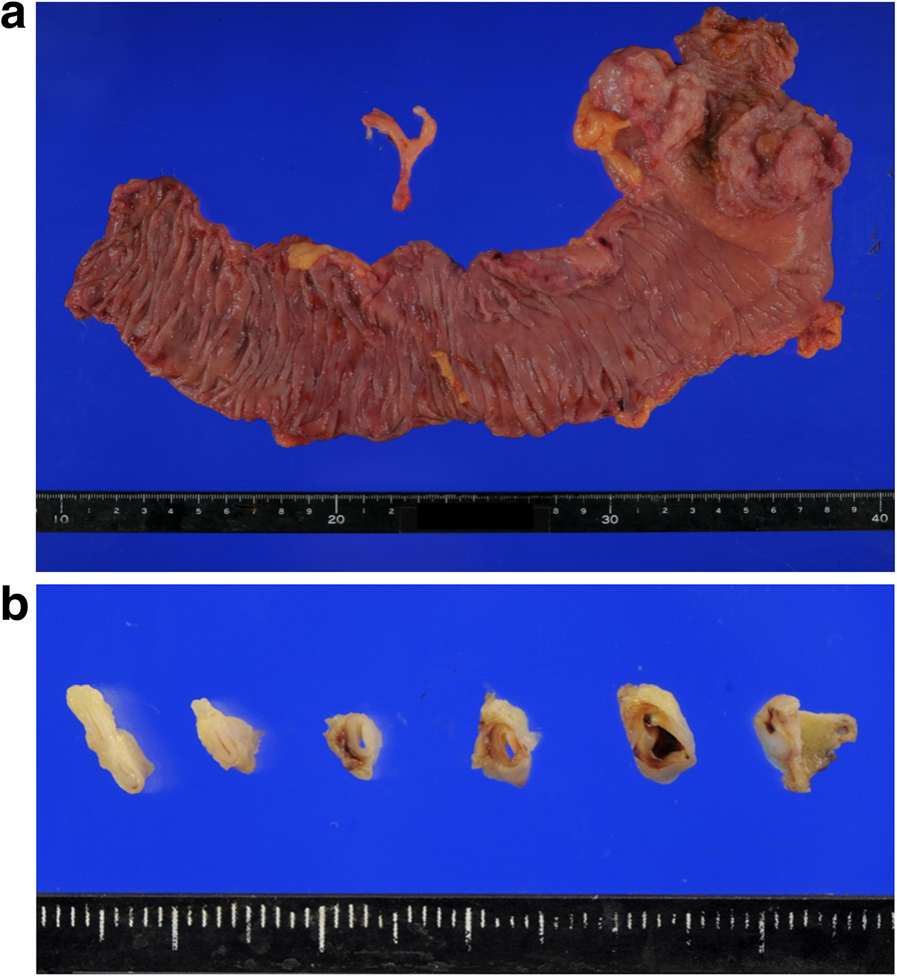
Resected Bormann type-2 cecal cancer specimen and shrunken middle colic artery aneurysm. **(a)** Both cecal cancer and aneurysm. **(b)** Aneurysm only.

The patient was placed in the modified lithotomy position under general anesthesia. First, a 12-mm trocar was placed using an open approach, and the abdominal cavity was explored with a 5-mm semi-flexible laparoscope (LTFVH; Olympus Medical, Tokyo, Japan). Pneumoperitoneum was induced and maintained at 8 mmHg with carbon dioxide gas. Three 5-mm ports and a 12-mm port were inserted into the abdominal cavity. The MCA aneurysm was clearly identified using a laparoscope (Figure 2). As the terminal ileum and the right and transverse colon were attached to the retroperitoneum, we mobilized them using Harmonic ACE shears (Ethicon Endo-Surgery, Cincinnati, OH, USA). A 5-cm abdominal incision was made via the umbilicus. A small wound retractor (ALEXIS wound retractor S, Applied Medical, Santa Margarita, CA, USA) was introduced for extracorporealization of the colon. Radical right hemicolectomy with D3 lymph node dissection was performed, and the MCA was ligated and divided at the root of the artery. Then, ileocolic functional end-to end anastomosis was conducted using staplers. Figure 3 shows the resected cecal tumor and MCA aneurysm. Pathological examination of the tumor revealed moderately differentiated tubular adenocarcinoma of the cecum without evidence of lymph node metastasis and that of the MCA aneurysm showed mediolysis and hyalization with fragmented and degenerated elastic fibers that were compatible with an arteriosclerotic change. The postoperative course was uneventful, and the patient survived without recurrence.

### Discussion

The true incidence of MCA aneurysms is unknown; however, they account for ≤3% of all visceral aneurysms [4]. Diagnosis of this disease rarely occurs in asymptomatic patients; instead, it usually occurs in patients suffering from complications of aneurysm, and 90% of these complications involve aneurysm ruptures [5,6]. Although the true etiology of MCA aneurysms remains unknown, connective tissue disorders including Marfan syndrome, Ehlers-Danlos syndrome, fibromuscular dysplasia, hereditary hemorrhagic telangiectasia, Osler-Weber-Rendu disease, and Kawasaki disease are considered to contribute. However, the present patient had no history of these connective tissue diseases or synchronous multiple aneurysms. This case was an incidental finding of MCA aneurysm without symptoms when we performed the preoperative three-phase dynamic computed tomography for disease staging.

The available evidence suggests that active treatment should be initiated with aneurysms ≥2 cm in diameter in patients at high risk of rupture (for example, pregnancy, childbearing age, and post-liver transplantation) [7]. Currently, transcatheter embolization is the first procedure of choice in most patients with visceral aneurysms because it can be carried out under local anesthesia with minimal physical burden [8].

However, we were unable to perform embolization in the present patient because it could hamper blood flow to his colonic anastomosis. In any event, elective intervention by either embolization or ligation should be considered for an asymptomatic aneurysm [5].

In this case, we considered that resection of ileo-colic and right colic arteries for cecal cancer could change the hemodynamics of the middle colic artery to increase risk of growing aneurysm. Further, in case of need to treat the aneurysm, impeded blood flow of the remaining left-side colon could occur especially in very elderly person who bear a risk of arteriosclerosis. Therefore, we performed combined resection of cecal cancer and the aneurysm by laparoscopic surgery which resulted in good short-term outcomes.

As a less invasive alternative to open surgery, laparoscopic resection of visceral aneurysms has been espoused by some authors [9]. The CLASICC trial conducted by the UK Medical Research Council confirmed the efficacy of laparoscopic surgery for both colon and rectal cancer in terms of short-term outcomes. Furthermore, improved short-term outcomes with laparoscopic surgery do not compromise the long-term oncological outcomes [3]. Using the laparoscopic approach, we were able to easily obtain information on the entire abdominal cavity and to perform a number of procedures through incisions including mobilization of the colon.

Kotake reported that D3 dissection is significantly superior to D2 dissection in terms of overall survival for patients with T3 and T4 colon cancer [10]. Recently, Kanemitsu and colleagues reported excellent long-term outcome of D3 dissection in right hemi-colectomy from single-institute non-randomized retrospective study in Japan [11].

To the best of our knowledge, the present case is the first reported in the English literature to undergo simultaneous laparoscopic surgery for both cecal cancer and an MCA aneurysm. We believe the advantages of laparoscopic surgery may result in safe and less invasive treatment in these patients.

## Conclusions

Laparoscopic radical right hemicolectomy for cecal cancer and an MCA aneurysm was successfully performed in an elderly patient. Laparoscopic surgery offers good short-term outcomes with uneventful postoperative recovery. The laparoscopic approach to visceral aneurysms may be a useful treatment option in patients for whom transcatheter treatment is not feasible.

## Consent

Written informed consent was obtained from the patient for publication of this case report and any accompanying images. A copy of the written consent is available for review by the Editor-in-Chief of this journal.

## Abbreviations

acRCV: accessory right colic vein
GCT: gastrocolic trunk
MCA: middle colic artery
SMA: superior mesenteric artery
SMV: superior mesenteric vein

## References

[1] Jamieson G, Hoffmann DC (2006). Colectomy: colonic conduits: the anatomy of the colon. The anatomy of general surgical operations.

[2] Stanley JC, Fry WJ (1974). Pathogenesis and clinical significance of splenic artery aneurysms. Surgery.

[3] Jayne DG, Thorpe HC, Copeland J, Quirke P, Brown JM, Guillou PJ (2010). Five-year follow-up of the Medical Research Council CLASICC trial of laparoscopically assisted versus open surgery for colorectal cancer. Br J Surg.

[4] Hirokawa T, Sawai H, Yamada K (2009). Middle-colic artery aneurysm associated with segmental arterial mediolysis, successfully managed by transcatheter arterial embolization: report of a case. Surg Today.

[5] Tessier D, Abbas M, Fowl R, Stone W, Bower T, McKusick M (2002). Management of rare mesenteric arterial branch aneurysms. Ann Vasc Surg.

[6] Yoo BR, Han HY, Cho YK, Park SJ (2012). Spontaneous rupture of a middle colic artery aneurysm arising from superior mesenteric artery dissection: diagnosis by color doppler ultrasonography and CT angiography. J Clin Ultrasound.

[7] Hunsaker DM, Turner S, Hunsaker JC (2002). Sudden and unexpected death resulting from splenic artery aneurysm rupture: two case reports of pregnancy-related fatal rupture of splenic artery aneurysm. Am J Forensic Med Pathol.

[8] Tulsyan N, Kashyap VS, Greenberg RK, Sarac TP, Clair DG, Pierce G (2007). The endovascular management of visceral artery aneurysms and pseudoaneurysms. J Vasc Surg.

[9] Arca MJ, Gagner M, Heniford BT, Sullivan TM, Beven EG (1999). Splenic artery aneurysms: methods of laparoscopic repair. J Vasc Surg.

[10] Kotake K, Mizuguchi T, Moritani K, Wada O, Ozawa H, Oki I (2014). Impact of D3 lymph node dissection on survival for patients with T3 and T4 colon cancer. Int J Colorectal Dis.

[11] Kanemitsu Y, Komori K, Kimura K, Kato T (2013). D3 lymph node dissection in right hemicolectomy with a no-touch isolation technique in patients with colon cancer. Dis Colon Rectum.

